# A Scoping Review to Contribute to Knowledge About Culturally Adapting Interventions for Latino Family Caregivers of Persons Living With Dementia

**DOI:** 10.1177/10436596241256328

**Published:** 2024-06-03

**Authors:** Daria B. Neidre, Roxana E. Delgado, Kimberly S. Peacock, Luis P. Luy, Carole L. White

**Affiliations:** 1 UT Health San Antonio, TX, USA

**Keywords:** Latino, family caregiving, cultural adaption, scoping review, dementia

## Abstract

**Introduction:**

Few interventions have focused on Latino family caregivers to persons with dementia, addressing their unique needs. This review aimed to identify best practices in cultural adaptation to make recommendations for adapting interventions for Latino family caregivers of persons living with dementia.

**Method:**

This scoping review was conducted following the Joanna Briggs Institute Scoping Review guidelines, with findings extracted and summarized from 17 studies addressing cultural adaptation.

**Results:**

Frameworks guiding the adaptations were comprehensive, addressing cultural values and traditions and the social context faced by Latino family caregivers. Features of the adaptations included diverse teams of researchers and community members, including Latino family caregivers, to inform the integration of cultural values into the content, mode, and place of intervention delivery.

**Discussion:**

Culturally adapting evidence-based interventions will increase the number of available interventions for Latino family caregivers to persons living with dementia, thus reducing inequities in caregiver support.

## Background

The Latino/Hispanic population is the fastest growing segment of older adults in the United States (Garcia et al., 2017). The strong association between aging and dementia coupled with the elevated risk for dementia among Latinos point to a growing number of Latinos being affected (Wu et al., 2016). This in turn implicates more family/friends, defined as family caregivers, who are taking on the role of providing care and support for the person living with dementia. Both the increase in numbers and the significance of the condition underscores the growing impact of Alzheimer’s disease and related forms of dementia (ADRD) for Latino families.

Caregiver burden has been reported to be greater among Latino family caregivers compared with non-Latino caregivers. Latino caregivers typically spend more hours per week on caregiving activities compared with their non-Latino counterparts, utilizing fewer formal care services (e.g., personal care, case management, and homemaking; Fenton et al., 2022; Gallagher-Thompson et al., 2003). Specific to dementia family caregivers, Latino caregivers perform a greater number of personal care tasks for their family members than non-Latino caregivers and take on more responsibility as dementia progresses (American Association of Retired Persons & The National Alliance for Caregiving, 2020). Results from a cross-sectional study of 139 Latino family caregivers to persons living with dementia indicated that about one-third of caregivers scored as mildly depressed, 17% scored as severely depressed, and almost all (90%) reported caregiver burden, ranging from mild to severe (Luchsinger et al., 2015). Given the negative outcomes for Latino family caregivers, there is a need to further consider the role of culture in family caregiving and how it may impact caregiver experiences, including their understanding of the clinical condition and seeking a diagnosis, the caregiving role and who takes on this role, and the utilization of community support services (Balbim et al., 2019; Dilworth-Anderson et al., 2020; Falzarano et al., 2022; Zarzycki et al., 2022).

While a substantial number of interventions have been designed and tested to examine their impact on reducing caregiver burden and improving health outcomes among family caregivers to people living with dementia (Gilhooly et al., 2016; Shi et al., 2020), fewer interventions have focused specifically on Latino family caregivers. Llanque and Enriquez (2012) conducted a review to identify intervention studies that targeted Latino family caregivers of persons living with dementia and identified only 10 published studies. Of these, seven publications were from the Resources for Enhancing Alzheimer’s Caregiver Health (REACH) studies. One decade later, another review and meta-analysis of interventions for Latino family caregivers to persons living with dementia was undertaken (Dessy et al., 2022). In this review, the authors identified 23 studies, with six studies focused exclusively on Latino family caregivers. While the authors noted progress in adapting interventions specifically for Latino family caregivers, they recommend the need for further studies that are designed with a focus on addressing the sociocultural context of caregivers.

To contribute to the knowledge of how to culturally adapt or tailor interventions specifically for Latino family caregivers of persons living with dementia, there is a need to identify best practices used in adaptation, including the elements of the intervention that are targeted in the adaptation as well as relevant adaptation frameworks (Barrera et al., 2013). Few studies targeting Latino family caregivers to persons living with dementia have provided sufficient detail regarding the adaptation process. This review, therefore, draws from the wider literature on Latino family caregiving to provide a more complete understanding of the process. The overall aim of this review was to gather information pertaining to cultural adaptation to make recommendations to inform future research about culturally adapting interventions for Latino family caregivers of persons living with dementia.

## Method

A scoping review methodology was considered the best choice for reviewing the literature on cultural adaptation for Latino family caregivers. The review included both qualitative and quantitative observational studies to identify cultural aspects important to consider as well as intervention studies testing culturally tailored interventions for family caregivers. The scoping review was conducted following the Joanna Briggs Institute Scoping Review guidelines (Peters et al., 2020), following the Preferred Reporting Items for Systematic Reviews extension for Scoping Reviews (PRISMAScR) checklist (Supplemental Appendix 1).

### Step 1: Defining the Research Question

The scoping review addressed the following questions(a) what frameworks have been used in adapting interventions for Latino family caregivers; (b) what are the characteristics of the adaptation process; and (c) how have cultural values and beliefs important to Latino family caregivers been integrated into the adaptation process.

### Step 2: Identifying Relevant Studies

Although the terms Hispanic and Latino are often used interchangeably in research, throughout the review, Latino was used to denote persons living in the United States whose origins can be traced to the Spanish-speaking regions of Latin America (Carteret, 2011). The following inclusion criteria guided the search (a) Latino family caregivers are the focus of study, regardless of the condition of the person they are caring for; (b) the study provides information about values that are important to Latino caregivers, describing how these values could be integrated into a measure or an intervention for Latino caregivers; and (c) study describes process of adapting or tailoring an intervention for Latino caregivers. Studies were excluded if (a) Latino family caregivers were not the primary focus; (b) the process of cultural adaptation was not described in the intervention studies; and (c) non-English studies.

**Figure 1.**
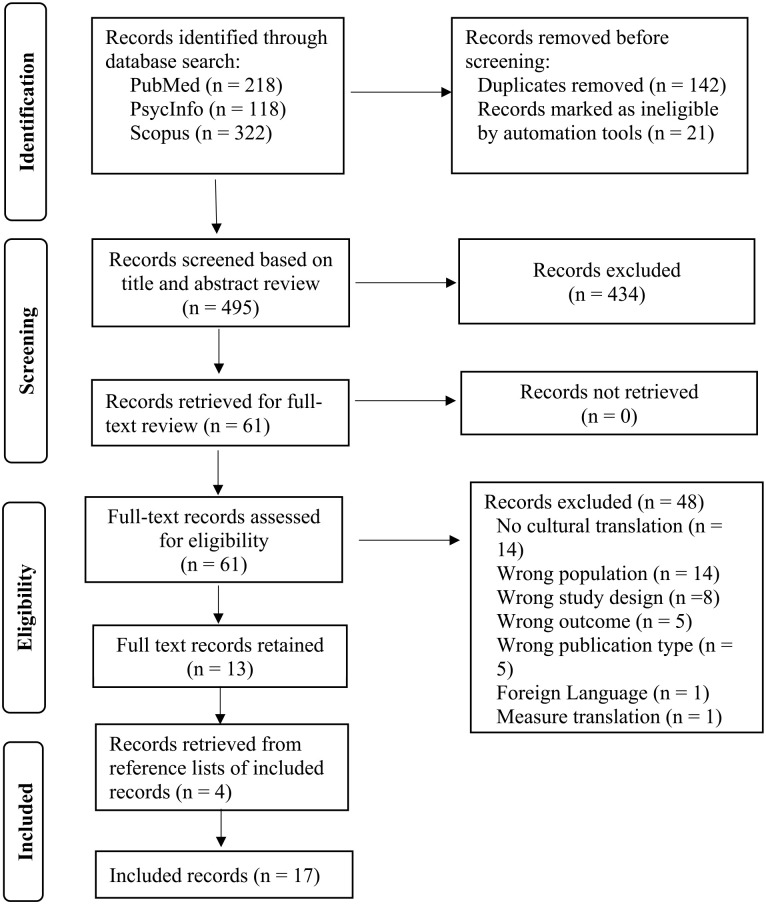
PRISMA Flow Diagram. *Source.* Adapted from Page et al. (2021).

The initial process was to identify the concepts for the search: Latino caregivers (Concept 1), cultural translation/adaptation (Concept 2), and interventions (Concept 3). The search string (Supplemental Appendix 2) was developed in collaboration with a health-sciences librarian and peer-reviewed by a second librarian according to the Peer Review of Electronic Search Strategies (PRESS) guidelines (McGowan et al., 2016). The PRESS review was completed on the PubMed search (January 1, 1990, to May 13, 2022) and determined sound logic, correct syntax, and no additional recommendations for adding or subtracting subject headings or keyword terms. The search was then expanded to PsycINFO (January 1, 1990, to May 13, 2022) and Scopus (January 1, 1990, to May 13, 2022), with final retrieval of 218 records from PubMed, 118 from PsycINFO, and 322 from Scopus (see Figure 1).

### Step 3: Selection of Studies

Results from all searches (*n* = 658) were imported into EndNote X9, duplicates removed (n=119) or marked as ineligible by automation tools (*n* = 21), and then imported into Rayyan (Ouzzani et al., 2016) where an additional 23 duplicates were identified and removed, leaving 495 records for abstract screening. Two team members independently screened the titles and abstracts, with disagreements resolved through discussion with the research team. A total of 61 studies were identified as eligible for full-text review and reviewed by two independent reviewers, with discrepancies resolved through research team discussion, with 48 studies excluded. Thirteen articles were retained with four further articles retrieved from hand-searching reference lists of eligible studies, leaving 17 studies.

### Step 4: Extracting and Charting the Results

The research team developed a data abstraction tool. Two reviewers independently abstracted data from two studies and discussed differences in data extracted. Following that, they abstracted the data from the remaining articles, meeting regularly to discuss abstracted data with the team, to resolve discrepancies.

### Step 5: Summarizing and Reporting the Results

Data were summarized based on the caregiver population, study design, whether an adaptation framework was used, and if so, the type of framework, distinguishing characteristics of the adaption, and study results.

## Results

Samples in the 17 retained studies included family caregivers to persons with cognitive decline and dementia, older adults, young children, stroke, diabetes, cancer, or depression, with cognitive decline or dementia being the most frequent condition (Table 1). About one-third of studies included the caregiver/care recipient dyad, one study included the family unit, with the remainder included only the family caregiver. Latino family caregivers were mostly classified as Mexican American, although several studies included caregivers from Puerto Rico, Cuba, and South America. About half of the studies were described as pilot studies (Beasley et al., 2014; Cruz-Oliver et al., 2016; Kajiyama et al., 2018; Mendez-Luck et al., 2019; Parra Cardona et al., 2012; Perrin et al., 2010; Santos et al., 2021; Valdez et al., 2013), most often utilizing a pre-post design to examine the effectiveness of an adapted intervention. Two studies described their protocol for cultural adaptation (Rush et al., 2015; Torres Blasco et al., 2022). The remaining studies were descriptive qualitative or quantitative designs with the objective of understanding the context of Latino family caregiving to guide the adaptation of an intervention or measure (Kao et al., 2010; Luna et al., 1996; Neary & Mahoney, 2005; Phillips et al., 1996; Rocha, 2019; Rote et al., 2019).

**Table 1.** Summary of Included Articles.

Article and year	Study purpose	Sample	Study design	Adaptation framework	Distinguishing features of adaptation	Main findings
Beasley et al. (2014)	Examine process of adapting SafeCare, an evidence-based parenting program, for Latino family caregivers	Latino mother caregivers of child under age 6 (*n*=28); Care providers (*n*=8)	Mixed-methods	Multi-level cultural framework for both surface (service materials) and deep structure (cultural factors, community, history)	Collaborative team including family caregivers; Contextualizing the content: translation, role of extended family, acculturation, traditional beliefs, relationship development, incorporating storytelling and proverbs, and considering racism, stereotypes, and discrimination	100% agreed that caregiving was easier since completing program and home safety was improved; Providers demonstrated improved knowledge and skills; Attrition 33%
Cardona et al. (2012)	Describe cultural adaptation of PMTO for Latino family caregivers	Latino family caregiver dyads (two parents) (*n*=12)	Pilot RCT comparing two culturally adapted versions of PMTO—CAPAS Original and CAPAS-Enhanced	Ecological Validity Model—cultural adaptation should impact specific areas of implementation such as linguistic appropriateness of curricula, appropriate methods of intervention delivery, cultural understanding of context	Qualitative investigation with 83 Latino immigrant parents which informed adaptation of the curriculum and study procedures; Community-based approach working with trusted community organizations who participated in delivery of intervention	High levels of satisfaction with both interventions; CAPAS-Enhanced participants emphasized importance of devoting more time to reflect on cultural themes of relevance to them; High retention and engagement for both interventions - 91%
Cruz-Oliver et al. (2016)	Develop a culturally tailored intervention for Latino family caregivers around EOL care	Latino caregivers to older adults from 2 sites in the United States and Puerto Rico (*n*=145)	Pilot study one-group pre- post-intervention design	No specific framework noted; needs assessment about EOL care with 45 hospice staff and observations in a hospice-guided culturally tailored intervention	Utilized a telenovela format, a genre popular with the Latino culture; Spanish and English versions of telenovela;	High satisfaction with intervention (92%) and reported learning new information (96%); Caregiver stress self-awareness and willingness to accept professional help improved significantly after intervention
Gonyea et al. (2016)	Evaluate effectiveness of Circulo de Cuidado in improving Latino family caregiver well-being	Latino family caregivers to persons with dementia (*n*=67)	RCT comparing culturally adapted version vs. control program	Broader social contextual framework used in tailoring the curriculum that acknowledged complexity of the caregiving situation, which is intensified by discrimination, linguistic isolation, and financial insecurity	Use of Spanish language and bilingual staff; Developed partnerships with local well-regarded organizations and interventions delivered at one of these sites; Groups were small, informal, and highly interactive; Recruitment and tailoring of intervention content based on values, beliefs, and experiences of Latino family caregivers	Compared with the control group, caregivers in the intervention group reported less distress about neuropsychiatric symptoms, greater sense of caregiver self-efficacy, and less depressive symptoms; Attrition 15%
Kajiyama et al. (2018)	Evaluate effectiveness of a telenovela in improving caregiver knowledge and decreasing stress and depression	Latino/His-panic caregivers to persons living with dementia (*n*=25)	Pilot study, one-group pre- post-intervention design	No specific adaptation framework noted but utilized a format for intervention delivery that is a popular Latino television genre with culturally tailored content	Telenovela format in Spanish (Latino actors), combining entertainment and psychoeducation; Online medium with DVD provided to those without internet access; Focus on strong cultural values and extended family working together	Significant decrease in stress, depression, and an increase in knowledge; Attrition 25%
Kao et al. (2010)	Discuss issues found in the pilot test of two instruments designed for use with MAs	Family caregivers of older MAs (*n*=22)	Pilot study testing utility of two Spanish-version instruments	No adaptation framework noted	Data from a community advisory council about their experiences as MA caregivers informed Spanish versions of scales; Bilingual and bicultural review panel of healthcare professionals with experience with MA immigrants	Issues identified in translation related to cultural equivalence and comprehension level that impact both the item and the response options
Luna et al. (1996)	Develop a cross-culturally equivalent definition and measure of familism for use with Anglos and MA caregivers	Bilingual MA caregivers (*n*=39) Spanish speaking MAs (*n*=30), English speaking bilingual MAs (*n*=30), Anglos (*n*=41)	Series of pilot studies to examine the Family Scale	No specific framework stated but used comparative framework for scale development to ensure aspects of familism expressed by both MA and Anglo caregivers	Detailed literature review on familism, family and caregiving, and belief systems; Qualitative and quantitative data from caregivers around familism	The concept of family exists in both cultures; Spanish-speaking caregivers had the highest familism scores, English-speaking bilingual caregivers the next highest scores, and Anglos the lowest scores
Mendez-Luck et al. (2019)	Design a culturally sensitive dyad-level diabetes intervention to improve glycemic control for older Latino adults with type 2 diabetes	Community-dwelling older adults with diabetes and their caregivers (*n*=39 dyads)	Pilot study, one-group pre-post intervention design	Guided by a framework that accounts for cultural beliefs about illness (Multigenerational Legacy of Diabetes Framework) and Sebern’s shared care construct, where dyad communicating about a shared situation leads to a shared understanding	Interviews with dyads followed by participant observation over 3 to 4 months to observe daily routines around diabetes care (*n*=12 dyads) informed intervention adaptation; Developed in Spanish and translated into English; Presented to 3 focus groups of caregivers and care receivers and revised based on their feedback	A1C values showed a trend of improved blood glucose control; Dyadic communication and self-rated health improved; Attrition 18%
Neary & Mahoney (2005)	Explore dementia caregiving among Latino caregivers to identify the influences of culture on caregiving	Latino ADRD caregivers of diverse origins (*n*=11)	Cross-sectional qualitative study	Leininger’s ethno-nursing approach	Team of nurse researchers and Hispanic Outreach Coordinator; Interviews conducted in Spanish and English	More similarities than differences in the multiethnic sample of Latino caregivers; Cultural influences—understanding of the condition; family-based home care based on intergenerational reciprocity, familial love, pride
Perrin et al. (2010)	Create a culturally sensitive educational supportive video-phone intervention for stroke caregivers	Stroke caregivers (65% Latino from Texas and PR) (*n*=61 dyads)	Pilot RCT (standard of care vs. intervention plus standard of care)	No adaptation framework noted	Caregiver guidebook developed in Spanish; Focus groups with clinicians and stroke caregivers evaluated the guidebook	High rate of caregiver satisfaction; Decreased strain and lower depression in the treatment group; Attrition 32%
Phillips et al. (1996)	Explore similarities in caregiving between MAs and Anglos caregivers with a focus on burden, familism, and quality of family caregiving	Pilot study 1: bilingual MA caregiver elder dyads (*n*=39); Pilot study 2: Anglos (*n*=41), Spanish-speaking MAs (*n*=29), Bilingual MAs (*n*=30)	Qualitative and quantitative pilot studies	Family Caregiving Dynamics Model—quality of caregiving for frail elders—focused on interactional, situational, interpersonal, and historical context of caregiving as an explanation for burden and poor-quality caregiving	Cross-cultural research team; Focus on the adequacy of instrumentation	Challenges of developing measures for bilingual participants; Experiences are not rooted exclusively in American or Spanish cultures
Rocha, 2019)	Assess and adapt a culturally sensitive health education program for family caregivers of persons with ADRD	ADRD Latina family caregivers and other Latino stakeholders (*n*=5 focus groups)	Cross-sectional, qualitative design	A Systematic Approach for Adapting Evidence-Based Behavioral Intervention Framework developed by the CDC	Adaptations to intervention based on findings from focus groups with family caregivers and stakeholders; Adaptations included changes to content, language to reflect the diverse roles of Latinas providing caregiving, and delivery format (activities at end to help caregivers relax and end on a positive note) The researcher has experience as a Latina family caregiver to her mother with ADRD	Need for intervention to include information about ADRD, the trajectory of care, and discussion on family dynamics; *Personalismo* is important in intervention delivery; Religion and spirituality; Building community and social networks
Rote et al. (2019)	Explore and describe the typology of family support in dementia caregiving which will inform types of interventions for sub-groups of Latinos	MA, English-speaking dementia family caregivers (=16)	Cross-sectional, qualitative design	No adaptation model noted	Importance of extended family involvement in Latino family caregiving; Family roles, and gender roles—need to recognize the familial role	Variability in family support availability for Latino caregivers; Caregivers with greater support reported larger family size, adaptable family members, help outside the family, and formalized processes for spreading caregiving duties across multiple persons
Rush et al. (2015)	Describe a multi-level partner-ship to implement a QOL intervention for a diverse sample of Latina breast cancer survivors and their caregivers	Multi-faceted team of researchers, clinicians, Latino cancer survivors, and family caregivers (*n*=13)	Description of adaptation of program and lessons learned	Adaptation of the program following principles of Community Based Participatory Research	Multi-level partnership among Latina survivors, caregivers, community-based organizations, clinicians, and researchers; Intervention reflects Latino values of *personalismo* and *familismo*; Attention to selection of scales that have good measurement properties in English and Spanish	Lessons learned: Importance of building relationships grounded in trust and mutual respect; Structure that allows input from all stakeholders at all phases of study; Democratic process in decision-making; Be clear about roles and responsibilities
Santos et al. (2021)	Develop linguistically and culturally appropriate adaptation of Memory Support System from English to Spanish and assess impact on caregiver outcomes	Spanish-speaking Hispanic adults with subjective cognitive decline (*n*=20) and support partners (*n*=16)	Pilot study	No adaptation framework noted	Professional forward and back translation of intervention materials and measures; Review by bilingual and bicultural professionals and volunteers unfamiliar with intervention with discrepancies resolved in consultation with initial translation services; Materials field tested with 4 Spanish- speaking community volunteers	Improved overall functional ability, depression, and anxiety for persons with cognitive decline; Less caregiver burden and anxiety; Attrition 17%
Torres Blasco et al. (2022)	Culturally adapt and integrate intervention for Latino families coping with advanced cancer and measure impact on QOL and spiritual well-being	Proposed sample size: Person with cancer and caregiver dyad (*n*=114 for quant phase and *n*=15 for qual phase for Phase Ib)	Protocol design	EVM used to adapt an intervention for a new cultural group; 7 dimensions to address: language, context, persons, metaphors, concepts, goals, and methods; CASA prescribes 4 phases for the adaptation process: formative, adaptation iterations, intervention, and measurement adaptation	Translate intervention into Spanish and eliminate spousal terms; Gather information from patients and informal caregivers for content and integrating Latinx cultural values to the CASA manual following dimensions outlined in EVM; Assess intervention with acceptability and feasibility questionnaire and semi-structured interviews (Phase Ib); Integration of the quantitative and qualitative findings to develop the fixed CASA protocol which will be examined for feasibility and acceptability (Phase IIa), followed by a pilot study (Phase IIb)	*N*/A
Valdez et al. (2013)	Culturally and linguistically adapt Keeping Families Strong for low-income Latino families and assess caregiver outcomes	Families (*n*=16) that include mother with depression, adult caregiver, and children	Pilot study, one-group pre-post intervention design	Adaptation based on the model of sociocultural processes affecting Latino families (differential acculturation between parents and children, cultural emphasis on family obligation and cohesion, parenting practices, stress, and isolation)	Adapted program, Fortalezas Familiares (Family Strengths); Held in a community agency rather than a mental health clinic; Families participated in a culturally representative meal intended to build group cohesion; Delivered in a multifamily group format (3–6 families) to create social support across the families, normalize the experience of depression, reduce stigma, and enhance learning and problem-solving through collaboration; Groups facilitated by mental health providers who are native Spanish speakers with substantial knowledge of Latino culture	Mothers reported decrease in depressive symptoms from pre- to post-test; Caregivers reported lower levels of depressive and anxiety symptoms; Mothers and caregivers reported a significant increase in social support, and improvement in family functioning; Attrition 19%

*Note*. PMTO = Parent Management Training-the Oregon model; CAPAS = culturally-adapted version of PMTO, Parent Management Training-the Oregon model; EOL = End of Life; RCT = Randomized Controlled Trial; MAs, Mexican Americans; ADRD = Alzheimer’s Disease and Related Dementias; PR = Puerto Rico; QOL = Quality of Life; EVM = Ecological Validity Model; CASA = Cultural Adaptation Process Model.

High levels of satisfaction and generally low attrition rates were reported in the pilot studies (Cruz-Oliver et al., 2016; Gonyea et al., 2016; Kajiyama et al., 2018; Mendez-Luck et al., 2019; Parra Cardona et al., 2012; Phillips et al., 1996; Santos et al., 2021; Valdez et al., 2013). A number of outcomes were examined across studies, and although conclusions are limited and related to weak designs, results are promising with the culturally adapted interventions showing improvement in caregiver outcomes across many domains. Studies reported improved caregiver knowledge (Beasley et al., 2014; Cruz-Oliver et al., 2016; Kajiyama et al., 2018), decreased stress, depression, anxiety, and caregiver burden (Cruz-Oliver et al., 2016; Gonyea et al., 2016; Kajiyama et al., 2018; Perrin et al., 2010; Santos et al., 2021; Valdez et al., 2013). Among other outcomes reported, Cruz-Oliver et al. (2016) reported that caregivers were more likely to accept professional help, Mendez-Luck et al. (2019) reported improved communication and self-rated health of caregivers and Valdez et al. (2013) reported improvements in social support and family functioning.

## Frameworks Guiding Cultural Adaptation

Beasley et al. (2014) utilized a multilevel cultural framework that addressed both surface and deep structures to adapt an evidence-based parenting program for Latino caregivers (Resnicow et al., 2000). Surface structure adaption was focused on cultural branding of the program, such as creating program materials to match the characteristics of their population. Deep structure adaptation focused on tailoring the intervention to include cultural values and tradition, aspects that may impact outcomes of the intervention. Both Parra Cardona et al. (2012) and Torres-Blasco et al. (2022) used an ecological validity model (Bernal et al., 1995) to guide their adaptation process. According to the model, adaption of an intervention for a new cultural group must address language, context, persons, metaphors, concepts, goals, and methods. Examples of how these aspects were integrated into the intervention process include translation, culturally relevant stories, and visual aids as well as the integration of Latino family values, traditions, and communication styles. Several investigators utilized frameworks which acknowledged the social context faced by Latino family caregivers, including poverty and racism, which compound the complexity of their caregiving situation (Gonyea et al., 2016; Mendez-Luck et al., 2019; Phillips et al., 1996; Valdez et al., 2013). Mendez-Luck et al. (2019) utilized the Multigenerational Legacy of Diabetes Framework (Scollan-Koliopoulos et al., 2005) that accounts for cultural beliefs about the illness to guide tailoring their dyadic intervention for Latino persons with diabetes and their family caregivers. Rocha (2019) utilized a framework developed by the Centers for Disease Control (CDC) which provides a systematic approach for adapting evidence-based interventions, with assessment, preparation, and implementation phases. While about half the studies did not report a framework for adaptation, some investigators utilized the results of interviews, focus groups, and participant observation to inform the adaptation (Mendez-Luck et al., 2019; Parra Cardona et al., 2012; Rocha, 2019; Rush et al., 2015; Torres Blasco et al., 2022).

## Distinguishing Features of the Adaptation

More than half of the studies utilized a team to guide the adaption, with Latino family caregivers participating on the team (Beasley et al., 2014; Rush et al., 2015) as well as a combination of researchers and service providers, with at least some members experienced in working with Latino families (Beasley et al., 2014; Gonyea et al., 2016; Kao et al., 2010; Luna et al., 1996; Neary & Mahoney, 2005; Parra Cardona et al., 2012; Rocha, 2019; Rush et al., 2015; Santos et al., 2021; Torres Blasco et al., 2022; Valdez et al., 2013). Investigators described utilizing modes of intervention delivery that would appeal to Latino family caregivers, including processes such as storytelling and proverbs that helped to contextualize the content. Both Cruz-Oliver et al. (2016) and Kajiyama et al. (2018) used a telenovela approach. Interventions were facilitated by bilingual facilitators, importantly those who had experience in Latino outreach (Kao et al., 2010; Neary & Mahoney, 2005; Rocha, 2019; Valdez et al., 2013). Place of delivery was also considered. Valdez and colleagues (2013) chose a community agency for the delivery of their mental health family intervention program rather than a clinic setting.

Investigators acknowledged that translation into Spanish was a necessary but insufficient aspect of cultural adaptation. Three studies focused on measurement, with the goal of establishing scales with good psychometric properties among Latino family caregivers to be used for measuring the impact of the adapted interventions (Kao et al., 2010; Luna et al., 1996; Phillips et al., 1996). Phillips et al. (1996) identified the challenges that exist in measurement for bilingual or Spanish-speaking caregivers as language equivalence does not equate to cultural significance. For example, the country of birth of the target population must be addressed when translating and adapting measurement scales as there are differences within the Spanish dialects that could impact the meaning and understanding of measurement scales (Kao et al., 2010).

## Integration of Cultural Values Into Adaptation

Lack of knowledge about a condition or disease, or cultural beliefs about the cause of an illness, such as dementia being attributed to old age, were recognized as important to address in adapting an intervention. Investigators collected information on caregiver beliefs about the conditions they were caring for through semi-structured interviews (Beasley et al., 2014; Mendez-Luck et al., 2019; Neary & Mahoney, 2005; Rocha, 2019; Rote et al., 2019). Using entertainment, psychoeducation, and culturally significant actors (i.e., religious leaders), these forms of delivery provided disease education to help change attitudes and build knowledge about the condition (Cruz-Oliver et al., 2016; Kajiyama et al., 2018). In adapting their dyadic diabetes intervention, Mendez-Luck et al. (2019) included discussion about the participants’ views about diabetes and activities related to diabetes care. In several studies focused on dementia family caregiving, foundational content about dementia, its causes, and its progression was added (Gonyea et al., 2016; Kajiyama et al., 2018; Rocha, 2019). Addressing cultural beliefs was associated with positive effects in the satisfaction, helpfulness, and acceptance of the adapted interventions (Beasley et al., 2014; Kajiyama et al., 2018; Mendez-Luck et al., 2019). Addressing knowledge and cultural beliefs helped Latino caregivers become more aware of burden and more open to receive professional support (Cruz-Oliver et al., 2016).

Semi-structured interviews were used to probe family involvement in the context of program engagement, gender beliefs, obligations to parents and own families, social and family contexts of caregiving, and improving communication (Beasley et al., 2014; Neary & Mahoney, 2005; Rocha, 2019; Rote et al., 2019). Investigators described interventionist training to build understanding of the importance of family within the Latino culture, incorporate the extended network directly and indirectly into services, and ensure mastery of delivery of the core components of the intervention (Beasley et al., 2014; Parra Cardona et al., 2012). Gonyea et al. (2016) adapted their content based on the cultural value of familism to include greater sensitivity in how a sense of family was experienced, internalized, and expressed by participants. Torres Blasco et al. (2022) integrated training on improving communication skills for couples and improving family communication dynamics, including adaptations for non-spousal caregivers by eliminating spousal terms and using more generalized caregiving terminology. In several studies, adaptations specifically addressed improving family communication (Rush et al., 2015; Torres Blasco et al., 2022; Valdez et al., 2013). For example, Rush et al. (2015) focused on improving communication skills among cancer survivors and their caregivers by emphasizing the impact of the diagnosis on everyone in the family and valuing the family unit over individual interests.

Most reviewed studies targeted relationship development as a key aspect of adaptation (Beasley et al., 2014; Cruz-Oliver et al., 2016; Gonyea et al., 2016; Mendez-Luck et al., 2019; Neary & Mahoney, 2005; Parra Cardona et al., 2012; Rocha, 2019; Rote et al., 2019; Rush et al., 2015; Santos et al., 2021; Torres Blasco et al., 2022; Valdez et al., 2013). The integration of the cultural value of personalismo (a personal connection; Kelley et al., 2020) was seen through various strategies used by researchers to understand the nature of relationships, how and when to discuss personal information, remembering children or other family members, remembering important events and managing communication (Beasley et al., 2014). Investigators partnered with trusted community-based partners in the form of local Latino community agencies, advisory board members, consultants, and churches and utilized bilingual or Latino research staff, interventionists or outreach coordinators (Gonyea et al., 2016; Mendez-Luck et al., 2019; Neary & Mahoney, 2005; Parra Cardona et al., 2012; Rocha, 2019; Rote et al., 2019; Rush et al., 2015; Santos et al., 2021). Investigators incorporated specific training for their interventionists to foster relationship-building through being present at interventions for a longer time to provide for mutual exchange of conversations, using interventionists with past experience of the disease, bilingual staff or volunteers, or through the use of cultural experts to help administer the intervention (Gonyea et al., 2016; Parra Cardona et al., 2012; Rush et al., 2015; Santos et al., 2021; Torres Blasco et al., 2022). Group interventions were conducted to encourage interaction among the researchers and group members (Beasley et al., 2014; Cruz-Oliver et al., 2016; Gonyea et al., 2016; Rocha, 2019; Valdez et al., 2013). Rocha (2019) emphasized the importance of activities at the end of the sessions to help caregivers relax and end on a positive note. Valdez et al. (2013) provided a culturally representative meal before the delivery of the intervention to build group cohesion. Investigators reported that participants felt “heard” when adaptation was centered to their personal needs (Gonyea et al., 2016; Mendez-Luck et al., 2019; Valdez et al., 2013).

## Discussion

**Figure 2.**
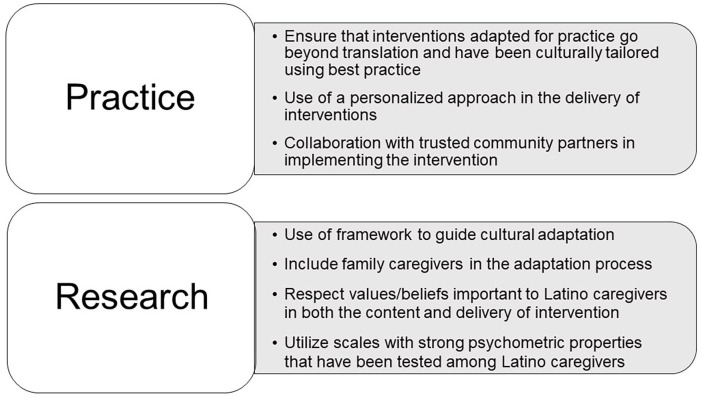
Practice and Research Recommendations Based on Findings From Scoping Review.

This scoping review aimed to describe the process of adapting interventions for Latino family caregivers, including frameworks guiding the adaptation, characteristics of the adaptation, and how cultural values important to Latino family caregivers were integrated into the intervention. While the number of interventions tailored for Latino family caregivers to persons living with dementia is growing, few studies have provided sufficient detail about the adaptation process, including guiding frameworks. This information is essential to inform future research in this area. Based on the findings from this scoping review, recommendations are made to guide both practitioners and researchers (Figure 2).

## Practice and Research Recommendations

Adapting a supportive care intervention for the practice setting requires more than Spanish language translation; it is imperative that the selected intervention for Latino family caregivers has been adapted using best practice. Modifications to the delivery and content of the intervention to include cultural elements may enhance participation among Latino family caregivers (Parker et al., 2023). The reviewed studies emphasized the importance of a personalized approach, including partnerships with trusted community organizations and individuals who have experience with Latino family caregivers. Delivery of the program necessitates an understanding among practitioners who implement the program of the cultural nuances and care preferences of Latino family caregivers. Translation of an intervention into practice could utilize community health workers (CHWs)/promotores in delivering the intervention (Varma et al., 2022). Trust is paramount, and as trusted members of their respective communities, CHWs can play a significant role in working with the team to develop trust with Latino family caregivers to persons living with dementia.

The review also offers recommendations to guide research. The first recommendation concerns the use of a framework to guide the adaptation. About half the studies did not describe a framework that guided their adaptation process and very few within the context of family caregiving for persons living with dementia. Frameworks help to systematically identify the many dimensions that are important to consider in the adaptation process (Barrera et al., 2013). Dessy et al. (2022) identified the need for future research that addresses the psychosocial, cultural, and economic factors that impact Latino family caregivers. A framework can provide a more comprehensive approach to adaptation, ensuring that factors such as the above are considered in both the content and the delivery. Within the review, several frameworks that consider the broader social context and how this contributes to the complexity of caregiving for Latino family caregivers were identified. Frameworks such as the multilevel cultural framework (Beasley et al., 2014) and the Ecological Validity Model (Parra Cardona et al., 2012; Torres Blasco et al., 2022) can guide cultural adaptation of an intervention for family caregivers. Gonyea et al. (2016) used a social contextual framework that acknowledged the discrimination, isolation related to language, and financial insecurity experienced by Latino family caregivers in adapting their intervention, using case examples that integrated many of these themes.

A second recommendation relates to the methodology of the adaptation. Several studies described a community-based participatory approach in conducting their research (Beasley et al., 2014; Rush et al., 2015) while other investigators used the content from focus groups or interviews with family caregivers to inform their work. Including the voices of the target population of family caregivers in the adaptation process, through participatory-based research, will contribute to the feasibility and relevance of the adapted intervention.

A third recommendation concerns the content and delivery of the intervention, addressing values important to Latino family caregivers. The reviewed studies demonstrated the importance of integrating content that acknowledged and respected the cultural beliefs of Latino caregivers, including beliefs about the condition, familismo (importance of family) (Kelley et al., 2020), and personalismo. Within the context of dementia family caregiving, several surveys have reported limited knowledge among Latinos about risk factors for dementia and the consequences of the diagnosis (Cruz, 2013; Gelman, 2010), with early symptoms of Alzheimer’s, including memory loss, attributed to old age and assumed to be an inevitable part of aging. In adapting interventions, it may be necessary to address the issue of health literacy around dementia and its causes by adding content about dementia, its diagnosis, and trajectory. Consideration of the strong value of family among Latinos can inform the adaptation of future interventions. In a national survey, 82% of Latino family caregivers reported at least one other unpaid relative or friend helping with caregiving responsibilities (Evercare, 2008). While many interventions have focused on a primary caregiver, considering the strong emphasis on family among Latinos and the likelihood of more than one caregiver, future intervention research could expand the focus to target multiple family members.

Concomitant with the need for further research to develop culturally tailored interventions for Latino family caregivers of persons living with dementia, a fourth recommendation relates to measurement. There is a need for scales with strong psychometric properties among Latino family caregivers to reliably measure the impact of the intervention. Within the reviewed studies, few investigators described the reliability and validity among Latino family caregivers of their selected measures (Gonyea et al., 2016; Rush et al., 2015; Santos et al., 2021). Rush et al. (2015) described selecting PROMIS measures that follow a strict translation protocol for cross-cultural and language translation (Eremenco et al., 2005). Two studies included in this review (Kao et al., 2010; Luna et al., 1996) underlined the importance of measurement and issues of cultural equivalence, with a focus on familism. Kao et al. (2010) identified the pitfalls related to using Spanish-translated versions of a scale that has been developed and tested only with English-speaking caregivers, including issues both at the level of the item and the response options. In many studies, related to the lack of measures tested among Latino family caregivers, investigators have, of necessity, relied on translated scales, without evidence of cultural equivalence for this population (Gallagher-Thompson et al., 2001). More recently, several studies have been conducted to examine the issue of cultural equivalence among measures developed for family caregivers (Teresi et al., 2020a, 2020b), helping to address the gap in reliable and valid measurement for Latino family caregivers.

## Limitations

The review findings need to be considered in light of some limitations. Although we used a rigorous search strategy, screening, and extraction process, it is possible that some studies may have been missed. In line with the scoping review methodology and considering our research objectives, a quality appraisal was not conducted. Given the nature of the review, while we were able to describe the elements that were considered and the process of adaptation, there is insufficient evidence to comment on the added benefit of a culturally adapted intervention vs. an intervention that has not been culturally adapted. While the analyses and results were described for Latino caregivers in general, there is some evidence that subgroups of Latino family caregivers may approach caregiving with different values and beliefs, particularly around familismo. (Gelman, 2014; Martinez et al., 2022). It was not possible to conduct the analyses by sub-group related to missing information in the articles or insufficient numbers for certain groups, thus affecting the generalization of the recommendations.

## Conclusion

The challenges faced by Latino family caregivers to persons living with dementia are compounded by their limited access to Spanish-language materials and culturally sensitive resources (Alzheimer’s Association, 2021). Adapting evidence-based family caregiver interventions is a key to increasing access to resources for Latino family caregivers (Wu et al., 2016). The findings from this review provide recommendations for future research aimed at adapting interventions for Latino family caregivers with the goal of reducing inequities in health care access for this vulnerable group.

## Supplemental Material

sj-docx-1-tcn-10.1177_10436596241256328 – Supplemental material for A Scoping Review to Contribute to Knowledge About Culturally Adapting Interventions for Latino Family Caregivers of Persons Living With Dementia Supplemental material, sj-docx-1-tcn-10.1177_10436596241256328 for A Scoping Review to Contribute to Knowledge About Culturally Adapting Interventions for Latino Family Caregivers of Persons Living With Dementia by Daria B. Neidre, Roxana E. Delgado, Kimberly S. Peacock, Luis P. Luy and Carole L. White in Journal of Transcultural Nursing

sj-docx-2-tcn-10.1177_10436596241256328 – Supplemental material for A Scoping Review to Contribute to Knowledge About Culturally Adapting Interventions for Latino Family Caregivers of Persons Living With Dementia Supplemental material, sj-docx-2-tcn-10.1177_10436596241256328 for A Scoping Review to Contribute to Knowledge About Culturally Adapting Interventions for Latino Family Caregivers of Persons Living With Dementia by Daria B. Neidre, Roxana E. Delgado, Kimberly S. Peacock, Luis P. Luy and Carole L. White in Journal of Transcultural Nursing
