# Supplementary material for: A Scoping Review to Contribute to Knowledge About Culturally Adapting Interventions for Latino Family Caregivers of Persons Living With Dementia
Source: J Transcult Nurs. 2024 Jun 3;36(1):43–56. doi: 10.1177/10436596241256328 (PMC11645853; doi:10.1177/10436596241256328)
Supplement: sj-docx-2-tcn-10.1177_10436596241256328 – Supplemental material for A Scoping Review to Contribute to Knowledge About Culturally Adapting Interventions for Latino Family Caregivers of Persons Living With Dementia [file sj-docx-2-tcn-10.1177_10436596241256328.docx]

**Appendix 2: Search Strategy and String Applied to PubMed**

| **Search** | **Query** |
| --- | --- |
| #1 | Latino Caregivers – CONCEPT  ((“Caregivers”[MeSH] OR “care giver”[TIAB] OR “care givers”[TIAB] OR caregivers[TIAB] OR caregiver[TIAB] OR carer[TIAB] OR carers[TIAB] OR "caretaker"[TIAB] OR "caretaker s"[TIAB] OR "caretakers"[TIAB] OR “care giver”[OT] OR “care givers”[OT] OR caregivers[OT] OR caregiver[OT] OR carer[OT] OR carers[OT] OR "caretaker"[OT] OR "caretaker s"[OT] OR "caretakers"[OT])  AND  ("Mexican Americans"[Mesh] OR "Hispanic or Latino"[Mesh] OR hispanic*[TIAB] OR "boricua*"[TIAB] OR "chicana*"[TIAB] OR “chicano*”[TIAB] OR “latinoamerican*”[TIAB] OR "mexican american*"[TIAB] OR “Puerto Rican*”[TIAB] OR latino*[TIAB] OR latina*[TIAB] OR latinx[TIAB] OR hispanic*[OT] OR "boricua*"[OT] OR "chicana*"[OT] OR “chicano*”[OT] OR “latinoamerican*”[OT] OR "mexican american*"[OT] OR “Puerto Rican*”[OT] OR latino*[OT] OR latina*[OT] OR latinx[OT]) OR ((“hispano*”[TIAB] OR “latine*”[TIAB] OR “latin”[TIAB] OR “latinu*”[TIAB] OR "latin american*"[TIAB] OR “Latin America”[Mesh] OR "spanish speak*"[TIAB] OR “mexico*”[TIAB] OR "Mexico"[Mesh] OR “cuban*”[TIAB] OR “peruvian*”[TIAB] OR “dominican*”[TIAB] OR "brazilian*"[TIAB] OR "central american*"[TIAB] OR "costa rican*"[TIAB] OR "guatemalan*"[TIAB] OR "honduran*"[TIAB] OR “uruguayan*”[TIAB] OR “Argentina”[Mesh] OR “argentine”[TIAB] OR “argentinian”[TIAB] OR “argentinean”[TIAB] OR “panamanian*”[TIAB] OR “salvadorean*”[TIAB] OR “salvadoran*”[TIAB] OR “salvadorian*”[TIAB] OR "nicaraguan*"[TIAB] OR "south america*"[TIAB] OR "bolivian*"[TIAB] OR "chilean*"[TIAB] OR "Chile"[Mesh] OR "colombian*"[TIAB] OR "ecuadorian*"[TIAB] OR "paraguay*"[TIAB] OR "Paraguay"[Mesh] OR "venezuelan*"[TIAB] OR "Puerto Rico"[Mesh] OR "spanish america*"[TIAB] OR “spanish caribbean*”[TIAB] OR "mexican american*"[TIAB]) AND ("United States"[MeSH] OR "united state*"[TIAB] OR "north america*"[TIAB] OR "North America"[MeSH] OR "Midwestern United States"[Mesh] OR "Southeastern United States"[Mesh] OR "Southwestern United States"[Mesh] OR "Northwestern United States"[Mesh] OR “Appalachia*”[TIAB] OR “great lakes”[TIAB] OR “mid atlantic state*”[TIAB] OR “mid atlantic region*”[TIAB] OR “middle atlantic state*”[TIAB] OR “middle atlantic region*”[TIAB] OR “midwestern us*”[TIAB] OR “midwestern state*”[TIAB] OR “midwest state*”[TIAB] OR “midwest us*”[TIAB] OR “great plains”[TIAB] OR “heartland”[TIAB] OR “new england”[TIAB] OR “northeastern us*”[TIAB] OR “northeastern state*”[TIAB] OR “northeast state*”[TIAB] OR “northeast us*”[TIAB] OR “pacific northwest”[TIAB] OR “northwestern us*”[TIAB] OR “northwest us*”[TIAB] OR “northwestern state*”[TIAB] OR “northwest state*”[TIAB] OR “pacific state*”[TIAB] OR “southeast state*”[TIAB] OR “southeastern state*”[TIAB] OR “southeast region”[TIAB] OR “southeastern region”[TIAB] OR “southeast us*”[TIAB] OR “southeastern us*”[TIAB] OR “southern state*”[TIAB] OR “southern us*”[TIAB] OR “southwest state*”[TIAB] OR “southwestern state*”[TIAB] OR “southwest us*”[TIAB] OR “southwestern us*”[TIAB] OR “deep south”[TIAB] OR “black belt”[TIAB] OR “rust belt”[TIAB] OR “district of Columbia”[TIAB] OR “Washington dc”[TIAB] OR “Alabama”[TIAB] OR “Alaska”[TIAB] OR “Arizona”[TIAB] OR “Arkansas”[TIAB] OR “California”[TIAB] OR “Colorado”[TIAB] OR “Connecticut”[TIAB] OR “Delaware”[TIAB] OR “Florida”[TIAB] OR “Georgia”[TIAB] OR “Hawaii”[TIAB] OR “Hawai i”[TIAB] OR “Idaho”[TIAB] OR “Illinois”[TIAB] OR “Indiana”[TIAB] OR “Iowa”[TIAB] OR “Kansas”[TIAB] OR “Kentucky”[TIAB] OR “Louisiana”[TIAB] OR “Maine”[TIAB] OR “Maryland”[TIAB] OR “Massachusetts”[TIAB] OR “Michigan”[TIAB] OR “Minnesota”[TIAB] OR “Minneapolis”[TIAB] OR “Mississippi”[TIAB] OR “Missouri”[TIAB] OR “Montana”[TIAB] OR “Nebraska”[TIAB] OR “Nevada”[TIAB] OR “New Hampshire”[TIAB] OR “New Jersey”[TIAB] OR “New Mexico”[TIAB] OR “New York”[TIAB] OR “North Carolina”[TIAB] OR “North Dakota”[TIAB] OR “Ohio”[TIAB] OR “Oklahoma”[TIAB] OR “Oregon”[TIAB] OR “Pennsylvania”[TIAB] OR “Rhode Island”[TIAB] OR “South Carolina”[TIAB] OR “South Dakota”[TIAB] OR “Tennessee”[TIAB] OR “Texas”[TIAB] OR “Utah”[TIAB] OR “Vermont”[TIAB] OR “Virginia”[TIAB] OR “Washington”[TIAB] OR “West Virginia”[TIAB] OR “Wisconsin”[TIAB] OR “Wyoming”[TIAB] OR america*[TIAB] OR "Americas"[Mesh]))) |
| #2 | **Cultural Translation/Adaptation** **– CONCEPT**  (("Cultural Diversity"[Mesh] OR "Cultural Competency"[Mesh] OR "Cultural Characteristics"[Mesh] OR "Cross-Cultural Comparison"[Mesh] OR "Culturally Competent Care"[Mesh] OR "Acculturation"[Mesh] OR cultur*[TIAB] OR cultur*[OT] OR ethnic*[TIAB] OR ethnic*[OT]) AND ("Translations"[Mesh] OR translat*[TIAB] OR translat*[OT] OR "Adaptation, Psychological"[Mesh] OR adapt*[TIAB] OR adapt*[OT])) OR (“cultural adaptation”[TIAB] OR “cultural adaptation”[OT] OR “cultural translation”[TIAB] OR “cultural translation”[OT] OR “cultural equivalenc*”[TIAB] OR “culturally adapted”[TIAB] OR “culturally adapted”[OT]) |
| #3 | **Interventions – CONCEPT**  N/A |

Search hedge adapted for Latino/Hispanic terms. Source: Medical Library Association Latinx Caucus. Latinx/Hispanic US Population Search Hedges. July 20, 2021. MLA. https://scholarshare.temple.edu/handle/20.500.12613/6949
